# A PPARγ-dependent miR-424/503-CD40 axis regulates inflammation mediated angiogenesis

**DOI:** 10.1038/s41598-017-02852-4

**Published:** 2017-05-31

**Authors:** Aram Lee, Irinna Papangeli, Youngsook Park, Ha-neul Jeong, Jihea Choi, Hyesoo Kang, Ha-neul Jo, Jongmin Kim, Hyung J. Chun

**Affiliations:** 1 Department of Life Systems, Sookmyung Women’s University, 52 Hyochangwon-gil, Yongsan-gu, Seoul 140-742 Korea; 2 Yale Cardiovascular Research Center, Section of Cardiovascular Medicine, Yale University School of Medicine, New Haven, CT USA

## Abstract

Activation of the endothelium by pro-inflammatory stimuli plays a key role in the pathogenesis of a multitude of vascular diseases. Angiogenesis is a crucial component of the vascular response associated with inflammatory signaling. The CD40/CD40 ligand dyad in endothelial cells (EC) has a central role in promoting vascular inflammatory response; however, the molecular mechanism underlying this component of inflammation and angiogenesis is not fully understood. Here we report a novel microRNA mediated suppression of endothelial CD40 expression. We found that CD40 is closely regulated by miR-424 and miR-503, which directly target its 3′ untranslated region. Pro-inflammatory stimuli led to increased endothelial CD40 expression, at least in part due to decreased miR-424 and miR-503 expression. In addition, miR-424 and miR-503 reduced LPS induced EC sprouting, migration and tube formation. Moreover, we found that miR-424 and miR-503 expression is directly regulated by peroxisome proliferator-activated receptor gamma (PPARγ), whose endothelial expression and activity are decreased in response to inflammatory factors. Finally, we demonstrate that mice with endothelial-specific deletion of miR-322 (miR-424 ortholog) and miR-503 have augmented angiogenic response to LPS in a Matrigel plug assay. Overall, these studies identify a PPARγ-dependent miR-424/503-CD40 signaling axis that is critical for regulation of inflammation mediated angiogenesis.

## Introduction

Endothelial cells (ECs) have unique functions in vascular biology. They can modulate immune response and angiogenesis, and have a key role in the maintenance of vascular homeostasis through protection of the vascular wall from pathological stimuli^1, 2^. Endothelial dysfunction under conditions of inflammatory stress is known to contribute to the pathogenesis of various diseases, including atherosclerosis, rheumatoid arthritis and tumor growth^1, 3–5^. Pathological angiogenesis is thought to be a key process in the inflammation-induced development of such diseases^1, 3–5^. Thus, it is essential to define the molecular mechanisms underpinning EC activation and consequent angiogenic signaling mediated by inflammatory processes. CD40, a transmembrane receptor of the tumor necrosis factor (TNF) gene superfamily, is largely expressed on antigen presenting cells (APCs), including B cells, macrophages, and monocytes^6, 7^. While it has been shown to be expressed at low levels in ECs, pro-inflammatory stimuli such as tumor necrosis factor alpha (TNFα) and lipopolysaccharide (LPS) cause rapid induction of CD40 expression in ECs^8–10^. It has already been demonstrated that the CD40/CD40 ligand (CD40L) signaling pathway plays a pivotal role in the immune response in various cells^7, 11^. Indeed, CD40-CD40L interactions induce various angiogenic factors such as VEGF, suggesting a potential role of CD40 in inflammation-mediated angiogenesis^12^.

Peroxisome proliferator-activated receptor gamma (PPARγ) receptors, transcription factors belonging to the nuclear hormone receptor superfamily, have been demonstrated to have widespread expression in the vasculature, including in ECs, vascular smooth muscle cells, and monocytes/macrophages^13–15^. It is known that PPARγ has anti-inflammatory properties, making it an important factor in maintaining vascular homeostasis^16^. As such, pioglitazone, a PPARγ agonist, acts to reduce inflammation in ECs thereby improving endothelial dysfunction^14, 17–22^. In addition, it has been shown that PPARγ agonists inhibit EC tube formation *in vitro* and VEGF-induced angiogenesis *in vivo* ^23–25^. Although it was reported that PPARγ agonists can inhibit CD40 expression^16^, the mechanism of this PPARγ-CD40 signaling axis in ECs remains to be investigated.

In this study, we sought to define the molecular mechanisms that govern inflammation-induced endothelial activation. We demonstrate that endothelial CD40 expression is closely regulated by two microRNAs (miRNAs), miR-424 and miR-503, and this regulatory mechanism becomes disrupted by inflammatory stimuli. Furthermore, we found that PPARγ is a direct transcriptional activator of miR-424 and miR-503, and its activation by pioglitazone leads to suppression of CD40 expression and LPS-induced angiogenesis in a miR-424 and miR-503 dependent manner. Moreover, we demonstrate enhanced angiogenic response to inflammatory stimuli in mice with endothelial specific deletion of miR-322 (miR-424 ortholog) and miR-503. These findings demonstrate a novel mechanism by which PPARγ signaling can augment the expression of homeostatic miRNAs, and likely serves as a key rheostat modulating endothelial inflammatory response.

## Results

### Pro-inflammatory stimuli induce CD40 and inhibit miR-424 and miR-503 expression in ECs

**Figure 1:**
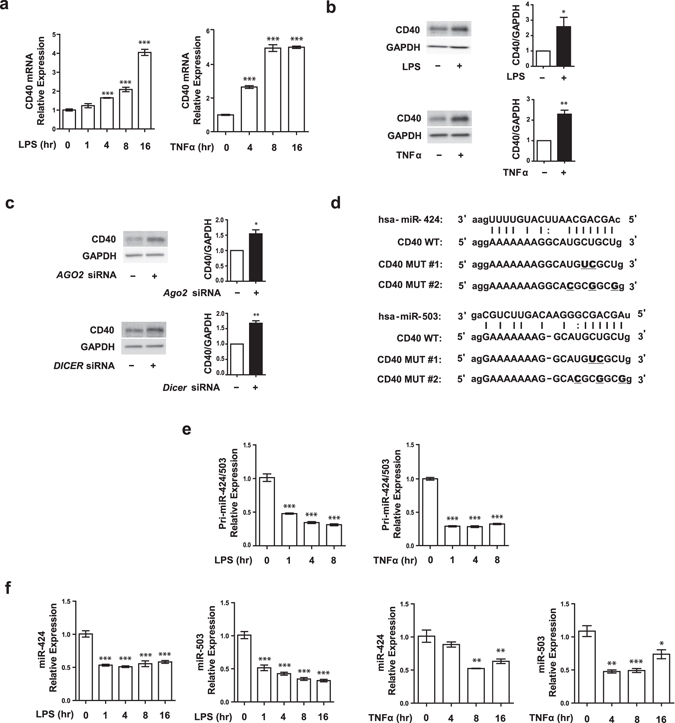
Pro-inflammatory stimuli upregulate CD40 and reduce miR-424 and miR-503 expression in HUVECs. (**a**) *CD40* mRNA expression in response to LPS (1 μg/ml) or TNFα (10ng/ml) in a time dependent manner. (**b**) CD40 protein expression in response to stimulation with either LPS (1 μg/ml for 24 h) or TNFα (10ng/ml for 16 h). (**c**) CD40 protein expression in HUVECs 72 h after *AGO2* or *DICER* siRNA transfection. (**d**) Predicted target sequences of CD40 3′ UTRs targeted by miR-424 and miR-503 and mutated sequences (MUT#1, #2) for disrupting miR-424 and miR-503 recognition sequence. (**e**,**f**) Quantitative PCR showing expression of the mature and pri forms of miR-424 and miR-503 in response to LPS (1 μg/ml) or TNFα (10ng/ml) in a time dependent manner. (**f**) *P < 0.05, **P < 0.01, ***P < 0.001 compared to controls. Error bars, s.e.m. Cropped gel images are shown; uncut gels are included in the Supplementary Information.

Previous studies have shown that pro-inflammatory stimuli lead to upregulation of CD40 expression in macrophages^26^. We assessed whether a similar mechanism exists in ECs. Treatment of human umbilical vein endothelial cells (HUVECs) with LPS (1 μg/mL) or TNFα (10 ng/mL) led to significantly increased CD40 mRNA and protein expression in a time dependent manner (Fig. 1a,b). Given prior studies implicating the involvement of miRNAs in inflammatory processes, as well as studies suggesting that CD40 itself is regulated by miRNAs in other contexts^27^, we investigated whether inflammation-induced CD40 expression is miRNA dependent. We found that subjecting HUVECs to knockdown of argonaute 2 (AGO2), a key protein of the RNA-induced silencing complex (RISC) complex^28–30^, or DICER, a key protein of miRNA processing, resulted in significant increase in CD40 protein levels (Fig. 1c), suggesting that baseline CD40 expression is miRNA dependent.

Next, we investigated which specific miRNAs may be involved in regulation of endothelial CD40 expression. Using a combination of target prediction software algorithms (TargetScan, PicTar, and microRNA.org), we found that miR-424 and miR-503, which have highly conserved seed sequences, were predicted to bind to the 3′ untranslated region (UTR) of CD40 (Fig. 1d). These two miRNAs are highly expressed in ECs and are known to have important roles in maintaining vascular homeostasis^28–32^. We next tested whether pro-inflammatory stimuli can affect endothelial miR-424 and miR-503 expression. We found significant decrease in both the immature pri-forms and the mature forms of miR-424 and miR-503 in response to either LPS or TNFα stimulation, suggesting that these miRs are transcriptionally suppressed in the context of endothelial response to inflammation (Fig. 1e,f).

### CD40 is directly targeted by miR-424 and miR-503

**Figure 2:**
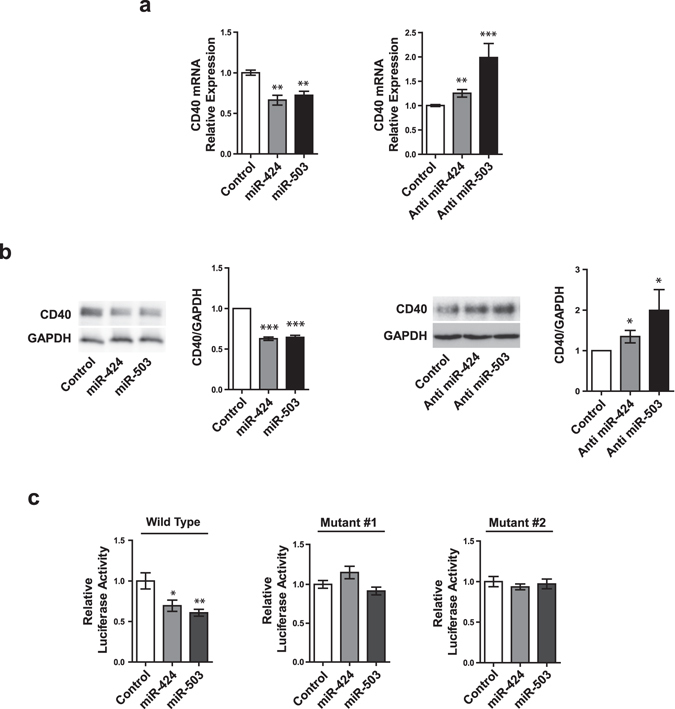
CD40 is regulated by miR-424 and miR-503 directly. (**a**) *CD40* mRNA expression in response to overexpression of miR-424 or miR-503 mimics, or inhibition of miR-424 or miR-503 with anti-miRs in HUVECs. (**b**) CD40 protein expression in response to overexpression of miR-424 or miR-503 mimics or inhibition of miR-424 or miR-503 with anti-miRs in HUVECs. (**c**) Effect of miR-424 or miR-503 overexpression on luciferase reporter containing either the wildtype of mutagenized 3′UTR of human *CD40* gene in HeLa cells. Luciferase activity data for constructs with the wild-type (WT) and mutants (#1 and #2) 3′ UTR constructs are shown. *P < 0.05, **P < 0.01, ***P < 0.001 compared to controls. Error bars, s.e.m. Cropped gel images are shown; uncut gels are included in the Supplementary Information.

We further investigated the relationship between miR-424/503 and CD40. Overexpression of miR-424 and miR-503 led to significant decrease in both the mRNA and protein expression of CD40, whereas inhibition of miR-424 and miR-503 using anti-miRs led to CD40 upregulation (Fig. 2a,b, Sup. Fig. 1a,b). We next determined whether miR-424 and miR-503 regulate CD40 expression via binding directly to the 3′UTR. Co-transfection of the wildtype CD40 3′UTR reporter construct with miR-424 and miR-503 mimics significantly reduced the reporter activity, while reporter activity in cells transfected with mutagenized CD40 3′UTR of two different types (mutant #1 and mutant #2) was unchanged by concurrent transfection with miR-424 and miR-503 mimics (Figs 1d and 2c). Combined, these results show that miR-424 and miR-503 directly regulate CD40 expression.

### CD40 inhibition attenuates LPS induced migration and tube formation in ECs

**Figure 3:**
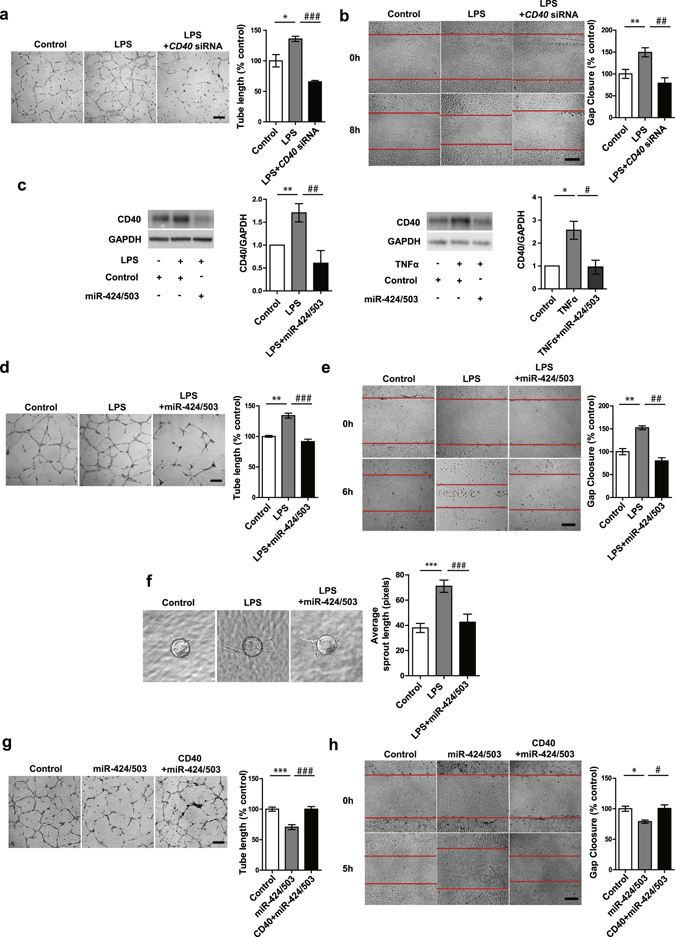
Inhibition of CD40 abrogates LPS-induced endothelial angiogenic response. (**a**,**b**) Tube formation and cell migration assay response to LPS (1 μg/ml) with or without *CD40* knockdown. Tubes were assessed at 8 h after cell plating. (**c**) CD40 protein expression in response to LPS (1 μg/ml), TNFα (10ng/ml) with or without miR-424 and miR-503 overexpression. (**d**,**e**) Tube formation and migration assay response to LPS (1 μg/ml) with or without miR-424/503 overexpression. Tubes were assessed at 8 h after cell plating. (**f**) Sprouting assay using miR-424/503 transfected HUVEC coated beads with or without LPS (1ug/ml) treatment. (**g**,**h**) Tube formation and migration assay response to miR-424/503 overexpression with or without CD40 overexpression at 5 h. *P < 0.05, **P < 0.01, ***P < 0.001 compared to controls. ^*#*^ *P* < 0.05, ^*##*^ *P* < 0.01, ^*###*^ *P* < 0.001 compared to LPS or miR-424/503. Scale bar, 200 μm. Error bars, s.e.m. Images are representative of three independent experiments. Cropped gel images are shown; uncut gels are included in the Supplementary Information.

Given the known endothelial angiogenic response when subjected to inflammatory stimuli, we next investigated the function of CD40 signaling in EC tube formation and migration assays. LPS significantly induced EC tube formation, an effect that was abrogated by concurrent CD40 knockdown (Fig. 3a). Furthermore, CD40 knockdown also significantly inhibited LPS-induced HUVEC migration (Fig. 3b). These findings indicate a key role for CD40 signaling in the regulation of angiogenic processes. The finding that miR-424 and miR-503 directly regulate CD40 led us to further investigate the effect of miR-424 and miR-503 overexpression on the regulation of pro-inflammatory stimuli-induced CD40 expression. Transfection of HUVECs with miR-424 and miR-503 mimics prior to treatment with LPS or TNFα led to significantly decreased CD40 protein expression (Fig. 3c). Moreover, overexpression of miR-424 and miR-503 significantly reduced LPS-induced EC migration and tube formation (Fig. 3d,e). In addition, we conducted sprouting bead angiogenesis assays, and found significant inhibition of LPS induced EC sprouting in cells that overexpress miR-424 and miR-503 (Fig. 3f). Lastly, we investigated whether CD40 overexpression could rescue miR-424 and miR-503 effects on EC tube formation and migration. We found abrogation of the effect of miR-424 and miR-503 overexpression by concurrent CD40 expression (Fig. 3g,h). Taken together, these findings support an important role for the miR-424/503-CD40 signaling pathway in pro-inflammatory stimuli-induced angiogenesis.

### Pioglitazone ameliorates LPS-induced angiogenesis through inhibition of CD40 expression

**Figure 4:**
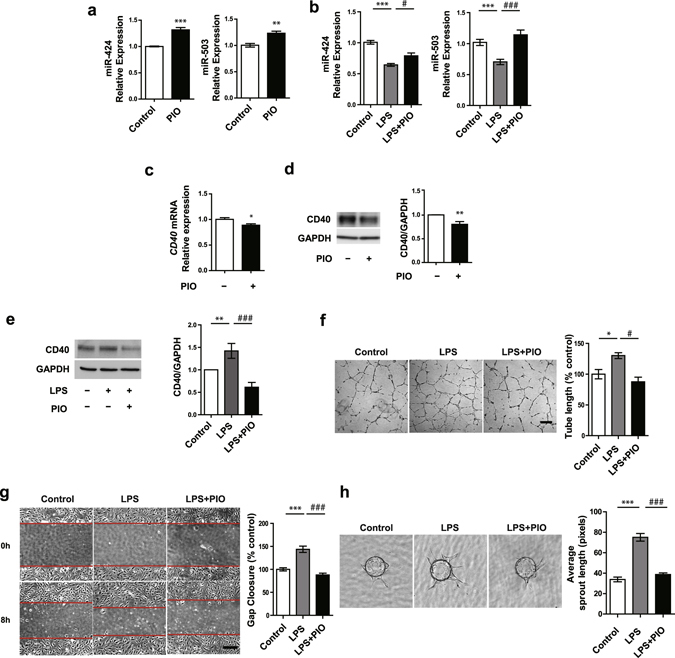
Pioglitazone ameliorates LPS-induced angiogenesis by regulation of CD40 expression. (**a**) Mature miR-424 and miR-503 expression in response to pioglitazone (10 μM) for 24 h. PIO, Pioglitazone. (**b**) Mature miR-424 and miR-503 expression in response to LPS (1 μg/ml) with or without concurrent stimulation with pioglitazone (10 μM) for 24 h. (**c**,**d**) CD40 mRNA and protein expression in response to LPS (1 μg/ml) with or without pioglitazone (10 μM) for 24 h. (**e**) CD40 protein expression in response to LPS (1 μg/ml) with or without pioglitazone (10 μM). (**f**,**g**) Tube formation and cell migration assay response to LPS (1 μg/ml) with or without concurrent stimulation with pioglitazone (10 μM) at 9 h. (**h**) Sprouting assay using HUVEC coated beads in response to LPS (1ug/ml) with or without pioglitazone (10 μM). *P < 0.05, **P < 0.01, ***P < 0.001 compared to controls. ^*#*^ *P* < 0.05, ^*###*^ *P* < 0.001 compared to LPS. Error bars, s.e.m. Images are representative of three independent experiments. Cropped gel images are shown; uncut gels are included in the Supplementary Information.

PPARγ has been found to significantly dampen inflammatory responses in ECs, and is a powerful pharmacological target for counteracting inflammatory diseases. In addition, a previous study has suggested indirect regulation of miR-424/503 expression by PPARγ via its induction of the G protein coupled receptor ligand apelin, which positively regulates miR-424 and miR-503^33^. To determine the molecular mechanism underlying the effect of PPARγ on pro-inflammatory stimuli-induced angiogenesis, we first examined the effect of pioglitazone, a PPARγ agonist, on miR-424 and miR-503 expression. Indeed, we found that treatment of HUVECs with pioglitazone resulted in a significant increase in miR-424 and miR-503 levels (Fig. 4a). In addition, we found that miR-424 and miR-503 levels were restored by pioglitazone when treated in conjunction with LPS (Fig. 4b). We also found that CD40 expression was decreased in response to pioglitazone stimulation (Fig. 4c,d), and that LPS-induced CD40 upregulation was reversed by treatment with pioglitazone (Fig. 4e). We further investigated whether pioglitazone can affect LPS-induced tube formation and migration in HUVECs. Pioglitazone reduced LPS-induced angiogenesis in HUVECs to baseline levels, as assessed by tube formation (Fig. 4f), migration (Fig. 4g), and sprouting angiogenesis assay (Fig. 4h). Taken together, these results suggest that pioglitazone attenuates LPS-induced angiogenesis by engaging the miR-424/503-CD40 signaling axis.

### PPARγ regulates miR-424 and miR-503 expression

**Figure 5:**
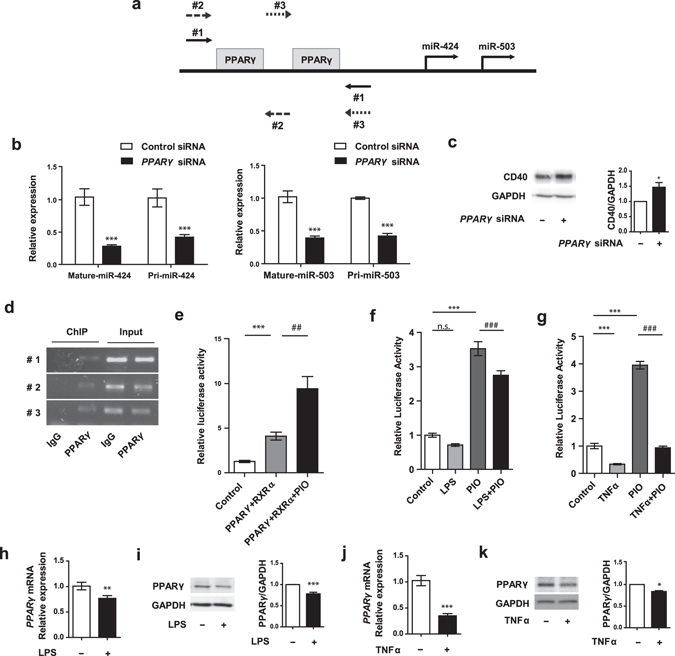
PPARγ signaling regulates miR-424/503 expression. (**a**) Predicted PPARγ binding sites on promoter region of miR-424 and miR-503 and the positions of PCR amplicons used in ChIP assays. (**b**) Mature and pri forms of miR-424 and miR-503 expression in response to *PPARγ* knockdown. (**c**) CD40 protein expression in response to *PPARγ* knockdown. (**d**) ChIP assay for investigating the binding sites of PPARγ on miR-424/503 promoter region. The ChIP pull-down PCR products (left) and the inputs (right) are shown. (**e**) Relative luciferase activity of HUVECs co-transfected with miR-424/503 promoter luciferase construct and PPARγ and RXRα with or without pioglitazone (10 μM) stimulation for 24 h. (**f**) *P < 0.05, **P < 0.01, ***P < 0.001 compared to controls. ^*##*^ *P* < 0.01, ^*###*^ *P* < 0.001 compared to PPARγ + RXRα or PIO. n.s.: not significant. Error bars, s.e.m. Cropped gel images are shown; uncut gels are included in the Supplementary Information.

We further investigated the relationship between PPARγ and miR-424/503. Our *in silico* analyses of the putative miR-424/503 promoter region (mapper.chip.org) identified two predicted PPARγ binding sites (Fig. 5a). We first examined the effects of PPARγ knockdown on miR-424/503 and CD40 expression. We found both the pri-forms and the mature forms of miR-424 and miR-503 were significantly downregulated by PPARγ knockdown (Fig. 5b). We also found CD40 expression was significantly elevated upon knockdown of PPARγ in HUVECs, further supporting involvement of PPARγ in the regulation of miR-424/503 mediated CD40 expression (Fig. 5c). Chromatin immunoprecipitation (ChIP) assays confirmed that PPARγ binds to the predicted binding sites of the putative miR-424/503 promoter region (Fig. 5d). Next, using a miR-424/503 promoter based luciferase reporter construct, we found significant induction of the luciferase reporter activity by overexpression of PPARγ and retinoid X receptor alpha (RXRα) (which forms a transcriptional complex with PPARγ)^34^. Moreover, the increased luciferase activity of HUVECs in response to PPARγ and RXRα overexpression was further augmented by concurrent treatment with pioglitazone (Fig. 5e).

We next investigated whether LPS and TNFα regulate miR-424 and miR-503 expression in a PPARγ dependent manner. Using a luciferase reporter construct driven by the minimal PPAR responsive element (PPRE), we found that LPS stimulation led to a trend towards decreased luciferase reporter activity at baseline, and significant decrease in PPRE reporter activity in response to pioglitazone (Fig. 5f). TNFα stimulation led to significant decrease in the PPRE luciferase reporter activity both at basal state and in conjunction with pioglitazone stimulation (Fig. 5g). Moreover, stimulation of HUVECs with either LPS or TNFα led to significant decrease in both the mRNA and protein levels of PPARγ, suggesting that decreased PPARγ expression in ECs may be a key mechanism by which miR-424 and miR-503 levels are decreased in response to inflammatory stimuli (Fig. 5h–k). Taken together, these results demonstrate that PPARγ is a key factor that controls miR-424 and miR-503 expression in response to endothelial inflammation.

### Mice with endothelial-specific deletion of miR-322 and miR-503 demonstrate increased angiogenesis in response to LPS

**Figure 6:**
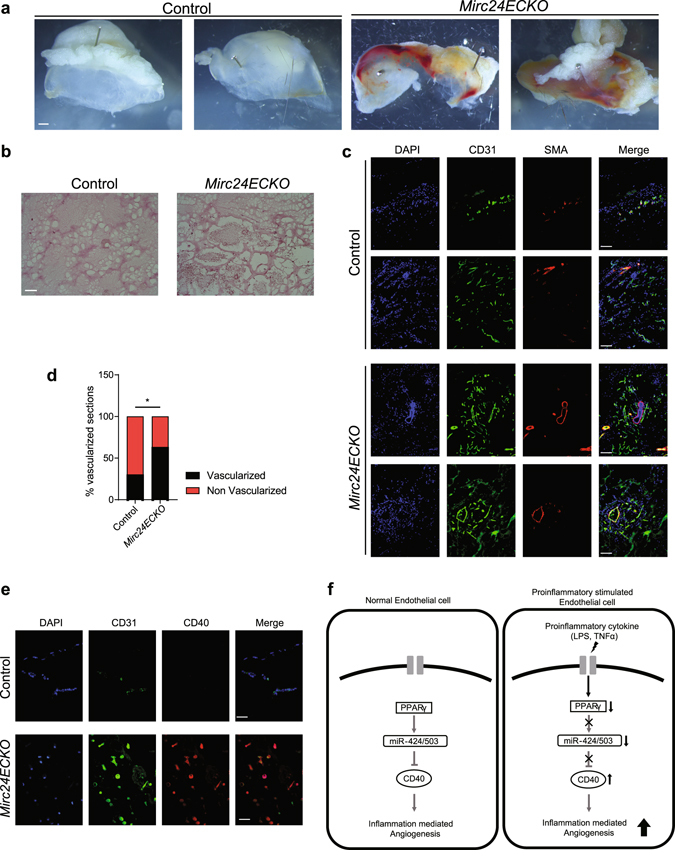
LPS induced angiogenesis is inhibited by *Mir24c in vivo*. (**a**) Whole mount images and (**b**) H&E stained sections of LPS-containing Matrigel implants, showing increased vascularization in mice lacking the Mir 24 cluster that includes miR-322 (mouse miR-424 ortholog), miR-503, and miR-351 (*Mirc24ECKO*). Scale bar: 100 μm. (**c**) Immunostaining of LPS-containing Matrigel implant sections for CD31 (green), SMA (red) and DAPI (blue) showing increased vascularization in *Mirc24ECKO*. Scale bar: 70 μm. (**d**) Quantification of vascularized/non-vascularized immunostained LPS-containing Matrigel implant sections in control and *Mirc24ECKO* mice. *P < *0.05*. (**e**) Staining for CD40 (red) in Matrigel implant sections along with CD31 (green) showing increased CD40 expression in infiltrating endothelial cells. Scale bar: 35 μm. (**f**) Schematic outlining the proposed endothelial mechanism.

To further investigate the relevance of our signaling paradigm *in vivo*, we evaluated the inflammatory angiogenesis response in mice with conditional, endothelial-specific deletion of miR-322 (mouse ortholog of miR-424) and miR-503 (*Mirc24fl/fl*;*Cdh5*-CreERT2, henceforth *Mirc24ECKO*). Mice with loxP sites surrounding the microRNA cluster 24 (which includes miR-322, miR-503, and miR-351) were crossed to mice with a tamoxifen inducible *Cdh5* Cre driver (Cdh5-CreERT2) (Sup. Fig. 2)^35, 36^. Adult *Mirc24ECKO* mice injected with tamoxifen displayed no overt phenotype up to 12 weeks after injection. Matrigel plugs containing LPS were implanted subcutaneously, and angiogenesis was assessed 7 days after injection. We found that *Mirc24ECKO* mice had significantly more neovascularization in the Matrigel plug in response to LPS compared to control mice, as demonstrated by increased blood content on gross assessment of the Matrigel plug (Fig. 6a), greater number of blood cell-filled vessels on H & E staining (Fig. 6b), and significantly increased number of CD31+/SMA+ mature vessels (Fig. 6c,d). This was in the context of increased CD40 expression in the ECs that infiltrated the Matrigel (Fig. 6e). Overall, these findings provide key *in vivo* genetic evidence supporting the downstream role of miR-424 and miR-503 in determining endothelial response to inflammatory stimuli.

## Discussion

Studies have reported that dysfunctional ECs are susceptible to pathogenic signals, resulting in various vascular diseases. Angiogenesis is a crucial component of the vascular response associated with activation of inflammatory signaling. The CD40/CD40L pathway is known to be involved in inflammatory response by promoting endothelial dysfunction. Here we identify a miRNA driven mechanism controlling CD40 signaling and show its involvement in the regulation of inflammation-mediated angiogenesis.

Four major conclusions can be drawn from the findings of this study: (i) CD40 and miR-424/503 are conversely regulated by proinflammatory stimuli in HUVECs. (ii) miR-424 and miR-503 directly target CD40 and inhibit inflammation-induced angiogenic phenotypes both *in vivo* and *in vitro*. (iii) PPARγ signaling regulates miR-424 and miR-503 expression in HUVECs. (iv) PPARγ agonism by pioglitazone reverses the downregulation of miR-424/503 and upregulation of CD40 caused by proinflammatory stimuli in HUVECs, thus ameliorating inflammation-induced angiogenesis (Fig. 6f).

Earlier studies investigating the regulation of angiogenesis have focused specifically on angiogenic genes and related signaling pathways. In recent years, research efforts have focused on the correlation between miRNA and angiogenesis in ECs. For example, it has been demonstrated that miR-15, 16, 20a, and 20b are anti-angiogenic miRNAs targeting VEGF mRNA^37, 38^, whereas the miR-17–92 cluster, miR-27b, and let-7f are thought to have pro-angiogenic properties^37, 38^. The miR-424 and miR-503 transcription unit is highly expressed in ECs and is known to be a key factor in maintaining homeostasis of these cells^28–32^.

Previous work has demonstrated that upregulation of miR-503 leads to inhibition of tumor angiogenesis through regulation of FGF2 and VEGFA^39^, while downregulation of miR-503 in ECs has been suggested to lead to improved angiogenesis in diabetes mellitus^40^. MiR-424 has been shown to target VEGF, VEGFR2 and FGFR1 and inhibit angiogenic activity in HUVECs^41^. In contrast, another study has shown that miR-424 may promote an angiogenic phenotype in hypoxic ECs^42^. Taken together, the studies to date suggest a context dependent role for miR-424 and miR-503 in ECs, and further studies are needed to fully elucidate the role of these highly-regulated miRNAs in endothelial function in health and disease.

In our current study, we examined the role of miR-424 and miR-503 in vascular inflammation and found that they control a key molecular mechanism involved in regulation of CD40 expression. Our findings demonstrate that miR-424 and miR-503 can inhibit pro-inflammatory-induced angiogenesis through direct targeting of CD40. Previous studies have implicated activation of CD40-CD40L interaction in atherosclerosis in ECs, smooth muscle cells, and macrophages^43^. CD40-CD40L signaling induces cell adhesion molecules, chemokines and tissue factor resulting in recruitment of lymphocytes in vascular endothelium^44–48^. It was also reported that soluble CD40L-induced CD40 activation has proangiogenic effects *in vitro* and *in vivo* ^12, 49, 50^. Our current findings identify a novel mechanism of CD40 signaling pathway regulation, and describe a PPARγ agonist-based pharmacologic strategy that can be used to modulate this signaling pathway.

It has been reported that the PPARγ agonist rosiglitazone suppresses CD40 expression and attenuates inflammation^16^. In addition, rosiglitazone was shown to attenuate inflammation in LPS-induced peritonitis^51^. However, the mechanism of PPARγ regulation of CD40 has remained elusive. In this study, we show that CD40 is upregulated in ECs subjected to pro-inflammatory stimuli, and demonstrate a novel PPARγ-miR-424/503-CD40 axis involved in its regulation. Our demonstration of PPARγ binding sequences in the miR-424/503 promoter region, in conjunction with our previous work identifying the transcription factor myocyte enhancer factor 2 (MEF2) as a key cis-acting factor involved in the regulation of miR-424 and miR-503^30^, point towards the need for future studies to investigate the potential synergy between these and other transcription factors.

The *Mirc24ECKO* mice represent a powerful genetic tool that can be used to further investigate the role of this miRNA cluster in endothelial function. The increased angiogenic response to LPS in the *Mirc24ECKO* mice, in conjunction with increased CD40 expression in ECs, support the hypothesis that unchecked endothelial response to inflammatory stimuli is an important driving force for our findings. Our studies do carry a number of limitations, including the artificial nature of the Matrigel plug assay, as well as potential effect of LPS on endothelial behavior such as maintenance of cell junction and permeability. Future investigation of these mice will provide greater insights into the role of these miRNAs in angiogenesis in both health and disease states.

In conclusion, we demonstrate that downregulation of miR-424 and miR-503 and subsequent upregulation of CD40 is associated with inflammation-induced angiogenesis. Furthermore, the demonstration that PPARγ directly regulates miR-424 and miR-503 expression identifies novel transcriptional targets of this pleiotropic gene that may in part explain its vaso-protective effects.

## Methods

### Animals

All animal experiments were conducted in compliance with the relevant laws and institutional guidelines and were approved by the Yale University Institutional Animal Care and Use Committee. *Mirc24* ^*fl/fl*^ and *Cdh5-CreERT2* mice have been previously described^35, 36^. All animals were maintained on a C57/Bl6 background. Mice were injected intraperitoneously with 1 mg of tamoxifen (Sigma Aldrich, T5648) dissolved in corn oil for five consecutive days. The mice were let to rest for an additional 14 days before experiments were conducted. All mice received tamoxifen.

### Cell Culture and Transfection

HUVECs (Lonza and Yale VBT Core) were cultured at 37 °C in a 5% CO2 incubator with EBM-2 basal medium supplemented with EGM-2 (Lonza) with 1% penicillin-streptomycin (Welgene). For experimental treatments, HUVECs (passages 3–7) were grown to 70% to 90% confluence. HeLa cells were cultured in DMEM (Welgene) supplemented with 10% fetal bovine serum (FBS, Gibco) and 1% penicillin-streptomycin (Welgene). SiRNA (Stealth siRNA, Invitrogen), miRNA mimics and anti-miRs (miRVanaTM, Ambion) were transfected using Lipofectamine RNAiMAX (Invitrogen) according to the manufacturer’s instructions. Cells were collected 72 h after transfection. HeLa cells were transfected with plasmids and miRNA mimics using Lipofectamine 2000 (Invitrogen). Pioglitazone was used at 10 μM for designated timepoints.

### Gene expression analysis

Total RNA was isolated with miRNeasy RNA isolation kit (Qiagen). Purified RNA was reverse transcribed using the TaqMan MiRNA Reverse Transcription Kit (Life Technologies). MiRNA quantitative reverse‐transcriptase PCR (qRT‐PCR) was performed using TaqMan Universal Master Mix II, no UNG (Life Technologies) and MiR-424 and miR-503 were detected with Taqman probes (Life Technologies). Data were normalized to the internal control small RNA (RNU19). For the mRNA, purified RNA was reverse transcribed using qPCRBIO cDNA Synthesis Kit (PCRBIOSYSTEMS). Quantitative RT‐PCR was performed using the qPCRBIO SyGreen Mix Hi-ROX (PCRBIOSYSTEMS) per manufacturer’s instructions. Ribosomal 18S was used as an internal control.

### Western Blotting

HUVECs were lysed with RIPA buffer (Gendepot) containing protease and phosphatase Inhibitor cocktail (Roche). Following this, centrifugation was performed at 13000 rpm, 4 °C, for 15 minutes. Protein concentrations were determined by Pierce BCA Protein Assay kit (Thermo Scientific). Equal amounts of total proteins were separated by sodium dodecyl sulfate–polyacrylamide gel electrophoresis, and transferred onto polyvinyl difluoride membrane (Millipore). Immunoblotting was performed with the primary antibodies specific to CD40 (1:3000, BD Bioscience), GAPDH (1:5000, Cell signaling), and PPARγ (1:5000, Santa Cruz). Immunodetection was accomplished using HRP-conjugated secondary antibodies (1:3000, Cell Signaling) and developed using an enhanced chemiluminescence detection method (Thermo Scientific).

### Luciferase Reporter Assay

For 3′UTR luciferase reporter assay, Human CD40 3′UTR (738p) that included predicted miR-424/503 binding seed sequences was cloned into NotI and XhoI sites of a psiCHECK-2 vector (Promega). The sequence TGCTGCT in the seed sequences of CD40 was mutated to TGTCGCT (Mutant #1), CGCGGCG (Mutant #2) using the QuikChange II Site-Directed Mutagenesis Kit (Agilent). HeLa cells were transfected with the luciferase reporter constructs and either miR-424, miR-503 mimics or negative control miRNAs using lipofectamine 2000 (Invitrogen). At 48hrs after transfection, the cells were lysed, and luciferase activity was measured using the Dual-Luciferase Reporter Assay kit (Promega) according to the manufacturer’s instructions. The human miR-424/503 promoter luciferase construct was previously described^31^. The PPAR responsive element (PPRE) promoter luciferase construct was a gift from Bruce Spiegelman (Addgene plasmid #1015). HUVECs were transfected with miR-424/503 promoter-luciferase, renilla-luciferase, PPARγ and RXRα constructs (Origene). Luciferase activity was quantified using the Dual-Luciferase Reporter Assay kit (Promega).

### EC tube formation assay

HUVECs were seeded on 6-well plates and transfected with siRNA or miRNA mimics using RNAi max (Invitrogen). The next day, 24-well plates were coated with 250ul of Matrigel Matrix (Growth Factor Reduced, phenol-free) (BD Bioscience) and incubated at 37 °C for 30 minutes. Meanwhile, transfected cells were trypsinized and seeded at 3 × 10^4^ cell per well on 24-well plates. The cells were then treated with LPS (Sigma, 1 μg/mL) and Pioglitazone (10 μM). Cells were observed under the microscope 8–9 h after being plated as designated for each experiment. WimTube (ibidi) was used to quantify the EC tube formation length.

### Cell migration assay

HUVECs were seeded at 2 × 10^5^ cells per well on 12-well plates and then, were scratched with a P200 pipette tip. HUVECs were incubated with 1% FBS starvation media containing LPS (1 μg/ml) or/and Pioglitazone (10 μM). The cells were allowed to migrate and the gap distance was captured with a camera equipped microscope at the designated timepoints. The width of the gaps was calculated using ImageJ.

### Fibrin gel bead assay

Cytodex 3 microcarriers (Amersham Pharmacia Biotech) were swollen in PBS for 3 h and sterilized by autoclaving. HUVECs were trypsinized and mixed with Cytodex 3 microcarriers at a concentration of 400 HUVECs per bead in 1 ml of EGM-2. Beads and HUVECs were incubated for 4 h and shaken every 20 min at 37 °C and 5% CO_2_. After incubation, beads with HUVECs were supplemented with 5 ml of EGM-2 and incubated overnight at 37 °C and 5% CO_2_. The following day, beads were washed twice with EGM-2 and resuspended in a solution of 2 mg/ml fibrinogen (Sigma-Aldrich) containing 0.15units/ml aprotinin (Sigma-Aldrich) at a concentration of 150 beads/ml. 500ul of fibrinogen/bead solution was added in a well containing 0.625units thrombin (Sigma-Aldrich). The fibrinogen/bead solution was allowed to clot for 5 min at RT and then at 37 °C and 5% CO_2_ for 15 min. During this time, fibroblasts were trypsinized and resuspended in EGM-2 and then plated on the top of the clot at a concentration of 20,000 cells per well. The medium was changed every other day. Average sprout length was analyzed using ImageJ.

### Chromatin Immunoprecipitation

HUVEC were plated on 100mm culture dishes and grown for 24 h before native protein-DNA complexes were crosslinked by treatment with 1% formaldehyde for 15 minutes. Simple ChIP Plus Enzymatic Chromatin IP kit (Cell Signaling) was used per the manufacturer’s protocol. Briefly, equal aliquots of isolated chromatin were subjected to immunoprecipitation with anti-PPARγ antibody or rabbit IgG control. PCR reactions of immunoprecipitated DNA were performed to validate PPARγ binding on the miR-424/503 promoter. PCR primers used: #1: FWD: GAGGTGGCTTTTTAGGGGT and REV: CGAGCCATCATGTCAGAAGT; #2: FWD: AGAGGCGTATTCTTTGGCTC and REV: TCCCTTCCAGATTGCTCTTG; #3: FWD: TCCAAGAGCAATCTGGAAGG and REV: TCTCCCAACATTTTGTTCCA. PCR products were separated by gel electrophoresis and visualized by SYBRsafe (Invitrogen).

### Lentivirus production

For CD40 overexpression in HUVECs, a lentivirus bearing CD40 was obtained from Origene. The Lenti-X HTX Packaging System (Clontech) with Lenti-X Concentrator was used to generate the lentivirus particles for *in vitro* cellular transduction.

### Matrigel plug angiogenesis assay

Liquid Matrigel (8–10 mg of protein/ml, Corning, 356231) was mixed at 4 °C with LPS (Sigma Aldrich, L2630) at a final concentration of 10 μg/ml and Heparin (Sigma Aldrich, H3393) at a final concentration of 20U/ml and injected subcutaneously (0.5 ml/injection) into the left and right groins of 5–6 month old *Mirc24* ^*fl/fl*^ or *Mirc24* ^*fl/fl*^ mir24fl/fl;*Cdh5*-CreERT2 female mice. The mice were sacrificed 7 d after injection and the plugs were fixed in 4% PFA for 2 h at RT, incubated in 30% sucrose and finally embedded in Tissue Tec OCT (Sakura). The Matrigel plugs were sectioned using a Leica CM1950 cryotome at 10 μm, and processed for immunohistochemistry or immunofluorescence.

### Immunohistochemistry and immunofluorescence

Matrigel sections were washed in 1x PBS, blocked for 1 h at RT in blocking buffer (1% BSA, 10% goat serum, 1xPBS) and incubated with anti-CD31 (BD Pharmigen, 553370), anti-smooth muscle actin conjugated to Cy3 (C6198, Sigma), and anti-CD40 antibody (R&D Systems, AF440) overnight at 4 °C. The next day the sections were incubated with Alexa Fluor secondary goat antibodies for 1 h at RT and counterstained with DAPI (Thermo Scientific) to visualize the nuclei. Imaging was performed using a Zeiss confocal microscope with a 10x objective. Vascularization was evaluated in 5 random fields per plug, 2 plugs per mouse, using ImageJ. H&E staining was performed using standard methods and visualized with a light microscope (Nikon Eclipse 80i).

### Statistical Analysis

All experiments were performed at least three times and analyses were performed with GraphPad Prism 5.0 software. When only two groups were compared, statistical differences were assessed with unpaired two-tailed Student’s t-test. Otherwise, statistical significance was determined using one-way analysis of variance followed by Bonferroni’s multiple comparison test. Relationships between variables were determined by the Pearson correlation coefficient. For the Matrigel plug analysis, two-tailed Fisher’s exact test was used to compare vascularization frequency in the mice of different genotypes. *P* < 0.05 was considered statistically significant.

## Electronic supplementary material

Supplementary Information
