# Supplementary material for: A PPARγ-dependent miR-424/503-CD40 axis regulates inflammation mediated angiogenesis
Source: Sci Rep. 2017 May 31;7:2528. doi: 10.1038/s41598-017-02852-4 (PMC5451412; doi:10.1038/s41598-017-02852-4)
Supplement: Supplementary file 1 — Supplementary Information [file 41598_2017_2852_MOESM1_ESM.pdf]

Supplementary Information for:

A PPARγ-dependent miR-424/503-CD40 axis regulates inflammation mediated angiogenesis

Aram Lee[1], Irinna Papangeli[2], Youngsook Park[1], Ha-neul Jeong[1], Jihea Choi[1], Hyesoo Kang[1], Ha-neul Jo[1], Jongmin Kim[1,2*], Hyung J. Chun[2*]

[1]Department of Life Systems, Sookmyung Women's University, 52 Hyochangwon-gil, Yongsan-gu, Seoul, 140-742, Korea, [2]Yale Cardiovascular Research Center, Section of Cardiovascular Medicine, Yale University School of Medicine, New Haven, CT USA

Content:

- Supplementary Figures 1-2
- Uncut blots

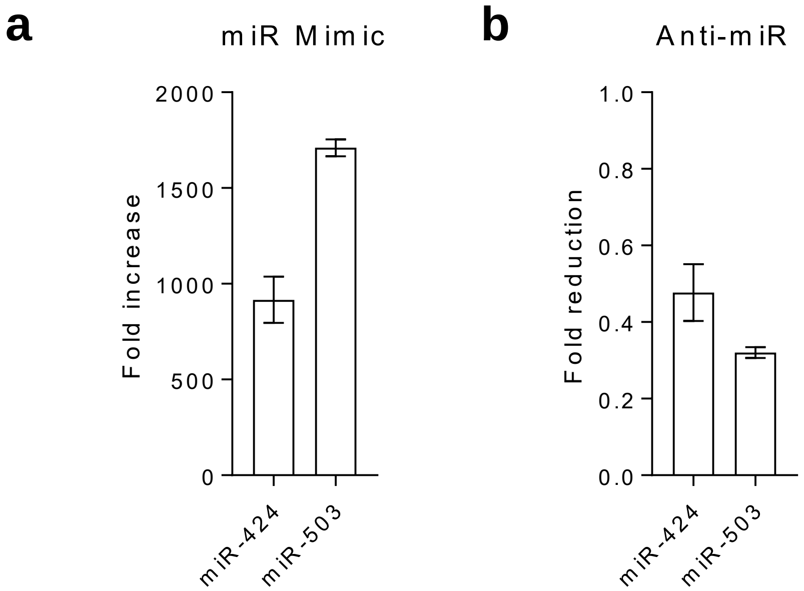

Supplementary Figure 1. Fold change of miR-424 and miR-503 in HUVECs transfected with either the respective miR-mimics (a) or anti-miRs (b).

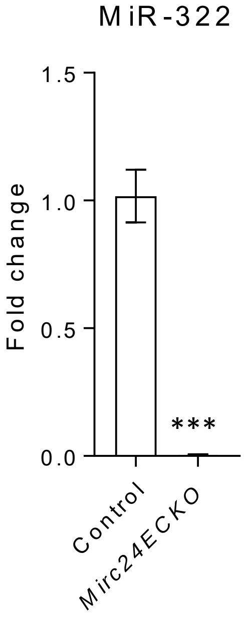

Supplementary Figure 2. Change in miR-322 (miR-424 mouse ortholog) expression in the lungs of tamoxifen injected Mirc24ECKO mice. *** $P < 0.001$.

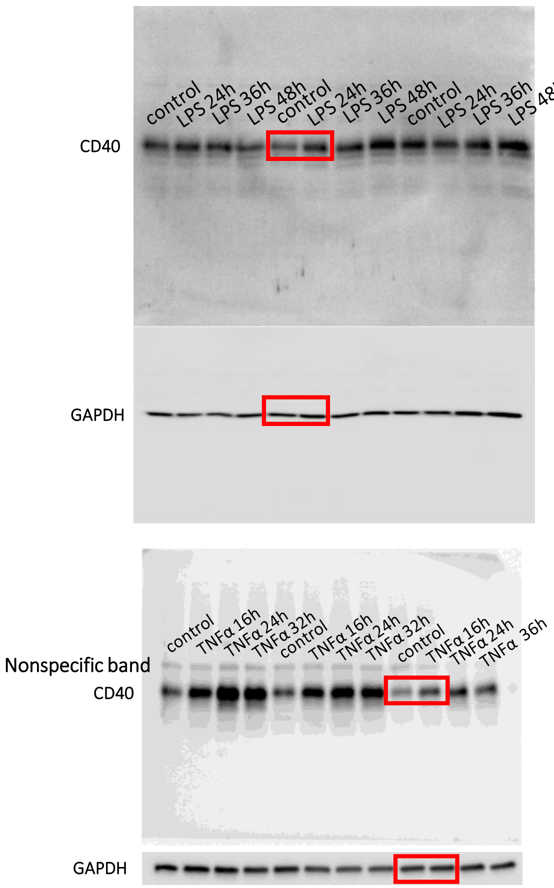

Fig. 1b

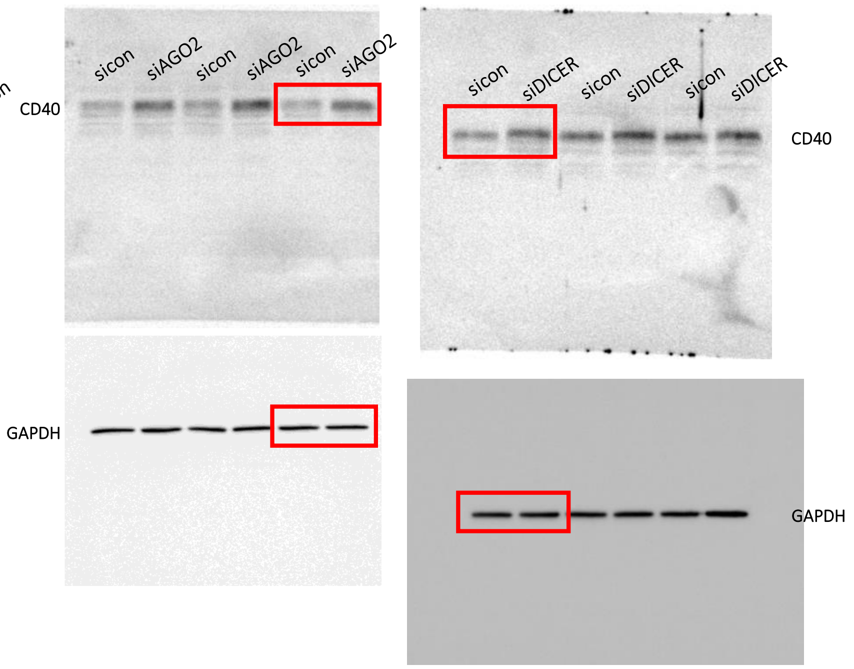

Fig. 1c

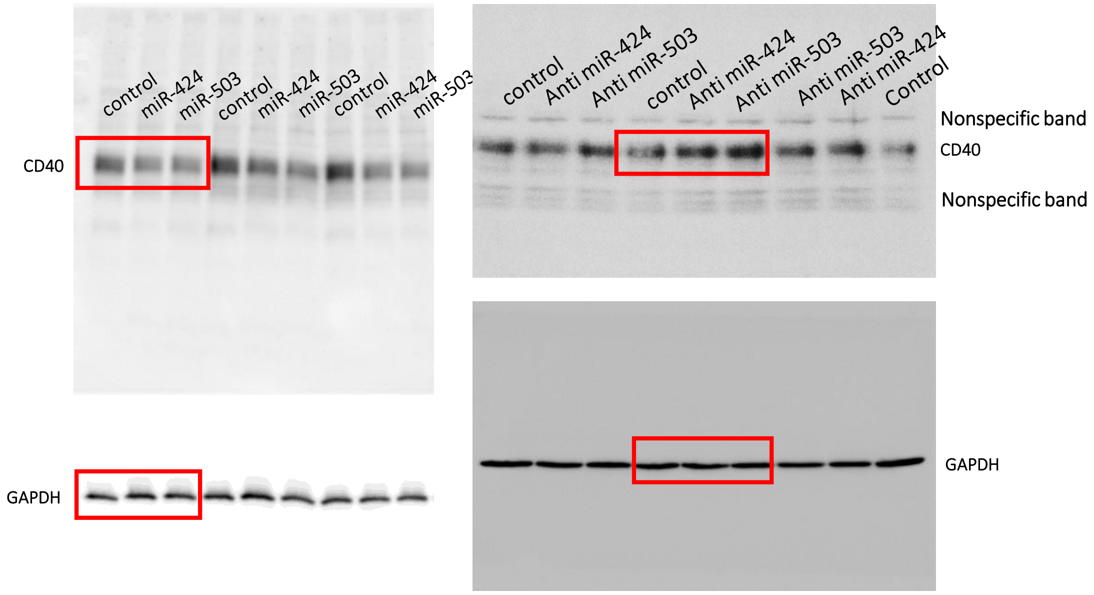

Fig. 2b

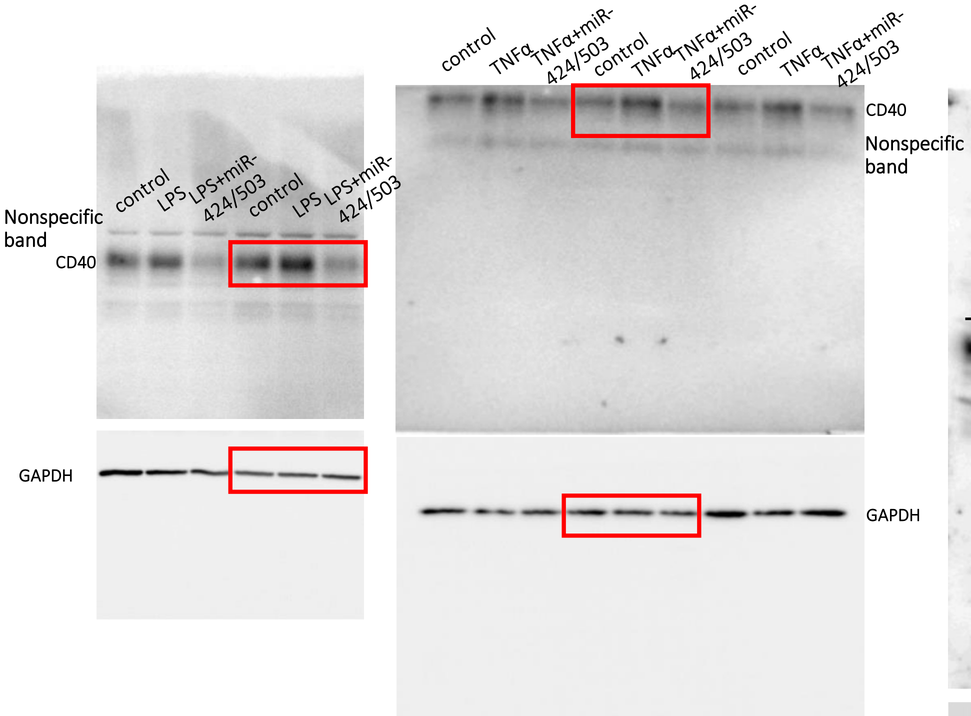

Fig. 3c

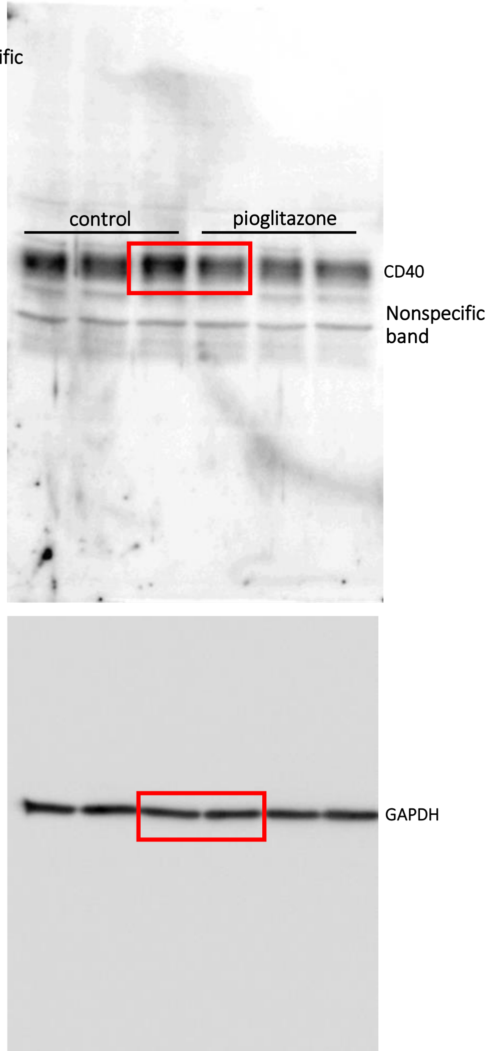

Fig. 4d

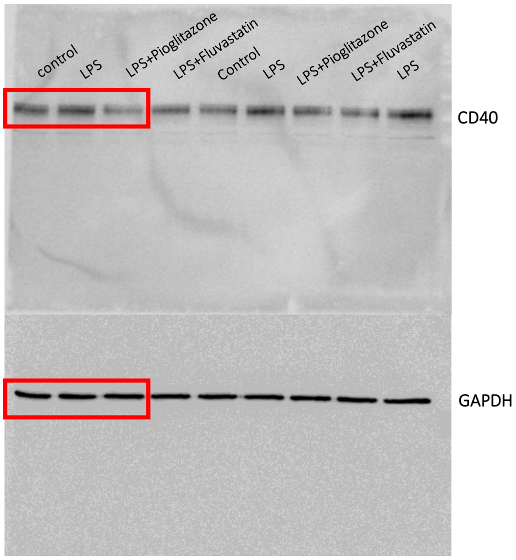

Fig. 4e

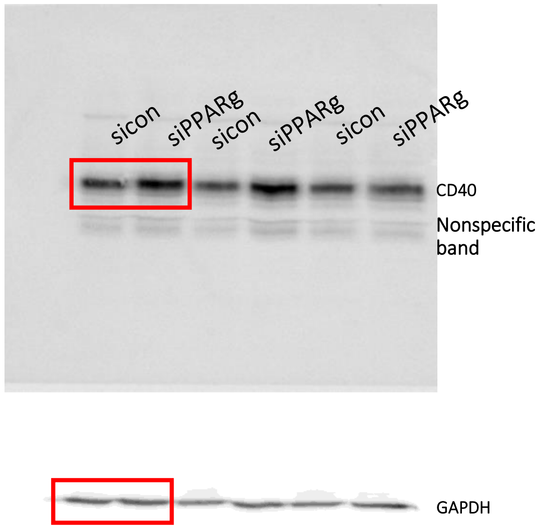

Fig. 5c

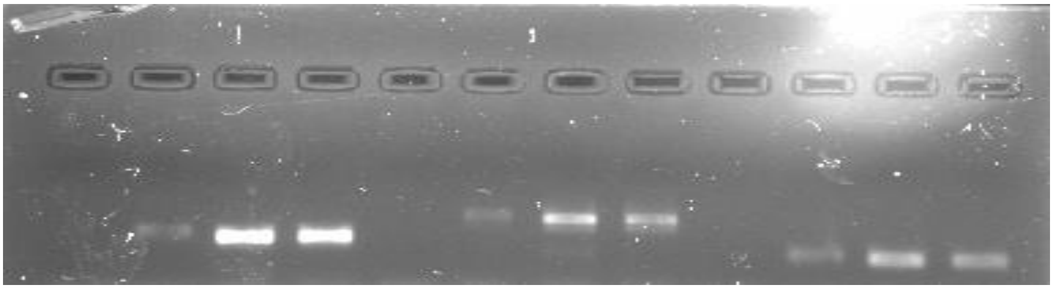

Fig. 5d

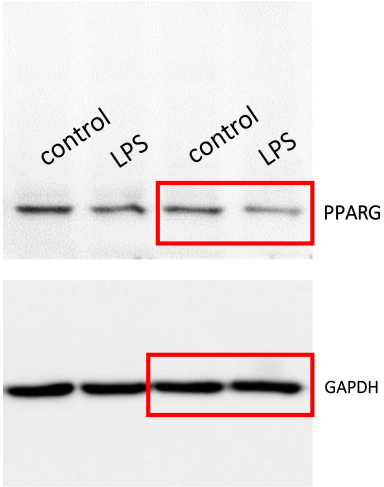

Fig. 5i

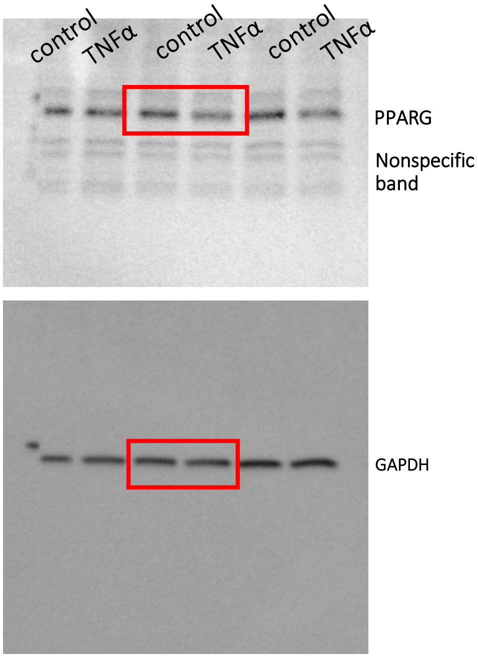

Fig. 5k
